# Standardized 4-step surgical approach to address atrial fibrillation during coronary artery bypass grafting in a patient with severely reduced left ventricular function: a case report

**DOI:** 10.3389/fcvm.2026.1966704

**Published:** 2026-10-28

**Authors:** Johannes Gökler, Philipp Szalkiewicz, Ilinca Damian, Jacqueline Illek, Gregor Sulzbacher, Andreas Zierer

**Affiliations:** Department of Cardiac, Vascular and Thoracic Surgery, Johannes Kepler University Linz, Kepler University Hospital, Linz, Austria

**Keywords:** ablation, atrial fibrillation, left atrial appendage, left posterior pericardiotomy, pulmonary vein isolation

## Abstract

Atrial fibrillation (AF) may result in haemodynamic deterioration and is particularly detrimental in patients with severely impaired left ventricular function. In this high-risk population, the maintenance or restoration of sinus rhythm (SR) in cardiac surgery are of paramount importance, warranting the use of all available surgical strategies to comprehensively address atrial fibrillation. We report the case of a 60-year-old man who underwent successful out-of-hospital cardiopulmonary resuscitation following an acute myocardial infarction. Coronary angiography revealed severe three-vessel coronary artery disease, accompanied by highly reduced left ventricular function. The patient had a history of symptomatic paroxysmal AF and had recently undergone electrical cardioversion to restore SR after the index myocardial infarction. In addition to surgical coronary revascularization via median sternotomy, a standardized four-step concomitant AF procedure was performed. This approach included bipolar radiofrequency ablation, exclusion of the left atrial appendage, transection of the ligament of Marshall, and left-sided posterior pericardiotomy. The patient was successfully weaned from cardiopulmonary bypass in SR and maintained stable SR throughout an 8-month follow-up period without AF recurrence. This case highlights the importance of systematically addressing AF during cardiac surgery, especially in high-risk patients with severely reduced ventricular function, where maintenance of sinus rhythm may have haemodynamic benefits.

## Introduction

Atrial fibrillation (AF) remains the most common sustained cardiac arrhythmia encountered in patients undergoing cardiac surgery and is associated with increased risks of stroke, heart failure, prolonged hospitalization, impaired quality of life, and reduced long-term survival (1).

Atrial fibrillation is particularly deleterious in patients with reduced left ventricular function undergoing coronary artery bypass grafting (2). In this context, in patients with heart failure and AF, restoration and maintenance of sinus rhythm has been associated with improvements in left ventricular ejection fraction, reverse remodeling, and, in selected cohorts, improved clinical outcomes including reduced heart failure hospitalizations and improved survival (2).

The 2024 ESC Guidelines introduced the AF-CARE pathway, emphasizing a patient-centred, multidisciplinary approach to AF management. Concomitant surgical AF ablation should be considered in appropriately selected patients undergoing cardiac surgery, with patient selection guided by AF characteristics, atrial and ventricular function, comorbidities, procedural risk, and the likelihood of maintaining sinus rhythm. Thus, surgical ablation should be individualized rather than routinely applied to all patients undergoing CABG (1).

Nevertheless, despite a growing body of evidence supporting surgical AF treatment, concomitant ablation remains substantially underutilized in daily practice, with reported implementation rates of less than 50% among eligible patients (3). The reasons for this discrepancy are multifactorial and include concerns regarding procedural complexity, additional operative time, increased ischemic duration, and uncertainty regarding lesion set selection (4). In a large analysis of 87,000 patients with atrial fibrillation undergoing isolated CABG, only 22% underwent concomitant surgical ablation, which was associated with better survival, improved quality of life and fewer hospitalizations (5).

Taken together, these findings suggest that maintenance of sinus rhythm is of particular importance in patients with severely reduced ventricular function undergoing CABG, where even small improvements in hemodynamic efficiency may translate into clinically meaningful effects.

In this context, we present a standardized 4-step surgical technique applied as a concomitant procedure during coronary artery bypass grafting (CABG) in a high-risk post-resuscitation patient, aiming to address AF in accordance with current guideline recommendations.

## Case presentation

A 60-year-old male patient was scheduled for surgical myocardial revascularization three weeks after successful resuscitation from an out-of-hospital cardiac arrest due to acute myocardial infarction, with return of spontaneous circulation (ROSC). The initial cardiac arrest rhythm was ventricular fibrillation. Initially, the patient´s medical history included paroxysmal atrial fibrillation and arterial hypertension. Prior to the index STEMI, the patient´s CHA₂DS₂-VASc score was 1 (hypertension) and the arterial fibrillation was clinically asymptomatic (EHRA symptom class I).

Coronary angiography revealed three-vessel disease with 80% left main coronary artery stenosis, proximal left anterior descending artery and mid-right coronary artery occlusions with collateralization, 70% first diagonal branch and 90% circumflex artery stenosis. No balloon angioplasty was performed in the setting of complex three-vessel coronary artery disease and the patient's hemodynamic stability after ROSC. Complete surgical revascularization was subsequently pursued following Heart Team discussion.

The post-infarction course was complicated by severely reduced left ventricular (LV) function resulting in a high-risk hemodynamic profile. The preoperative LVEF was 25%, with uniform hypokinesia on the first transthoracic echocardiogram after return of spontaneous circulation. Echocardiography revealed normal left ventricular dimensions, a slightly dilated left atrium, and normal mitral valve function. Following levosimendan therapy, the LVEF improved slightly to 30% prior to revascularization.

At this stage, severe left ventricular systolic dysfunction (LVEF 25%–30%) and the recent myocardial infarction resulted in a CHA₂DS₂-VASc score of 3 (hypertension, vascular disease, and heart failure).

Shortly after ROSC, the patient developed hemodynamically significant tachyarrhythmic AF requiring electrical cardioversion, which was transesophageal echocardiography guided.

Prior to the STEMI, the patient was receiving beta-blocker therapy for arrhythmia control and oral anticoagulation with a NOAC. NOAC therapy was bridged with therapeutic-dose LMWH prior to surgery. No antiarrhythmic medication other than beta-blockers was administered at any time.

Based on the patient's symptomatic paroxysmal AF, recent hemodynamically significant AF episode, severely impaired LV function, and the anticipated benefit of maintaining sinus rhythm, the multidisciplinary heart team considered concomitant surgical AF treatment appropriate in this individual patient. An institutional four-step approach was subsequently performed as an adjunct to complete coronary revascularization. We routinely perform this surgical approach via median sternotomy under cardiopulmonary bypass to address atrial fibrillation in non-atrioventricular valve surgeries in line with current guideline recommendations for concomitant AF surgery (Supplementary Video 1):

1. Pulmonary vein isolation and left atrial box lesion

Epicardial bipolar radiofrequency ablation was performed to achieve bilateral pulmonary vein isolation and creation of a left atrial box lesion set (Isolator Synergy EnCompass® clamp (AtriCure, Inc., Mason, OH, USA)). This was performed on cardiopulmonary bypass without left atriotomy, thereby avoiding additional cross clamp time, atrial incision and minimizing procedural complexity. (Figures 1A,B)

2. Left atrial appendage management (LAA)

Surgical exclusion of the left atrial appendage was performed after aortic cross-clamping. In the present case, resection was chosen instead of clip exclusion due to the concern for kinking of the graft and potential thrombus formation seen in transesophageal echocardiogram. The appendage was resected at its base (Figures 1C,D) and closed with a double-layer running polypropylene suture (Figure 1E).

3. Ligament of marshall transection (LOM)

The ligament of Marshall was divided to eliminate a recognized autonomic and arrhythmogenic structure contributing to the initiation and maintenance of atrial fibrillation.

4. Left-sided posterior pericardiotomy (LPP)

A posterior pericardial incision was created parallel to the phrenic nerve to facilitate postoperative drainage into the left pleural space (Figure 1F). This step was intended to reduce postoperative pericardial effusion and potentially decrease early postoperative atrial arrhythmias.

**Figure 1:**
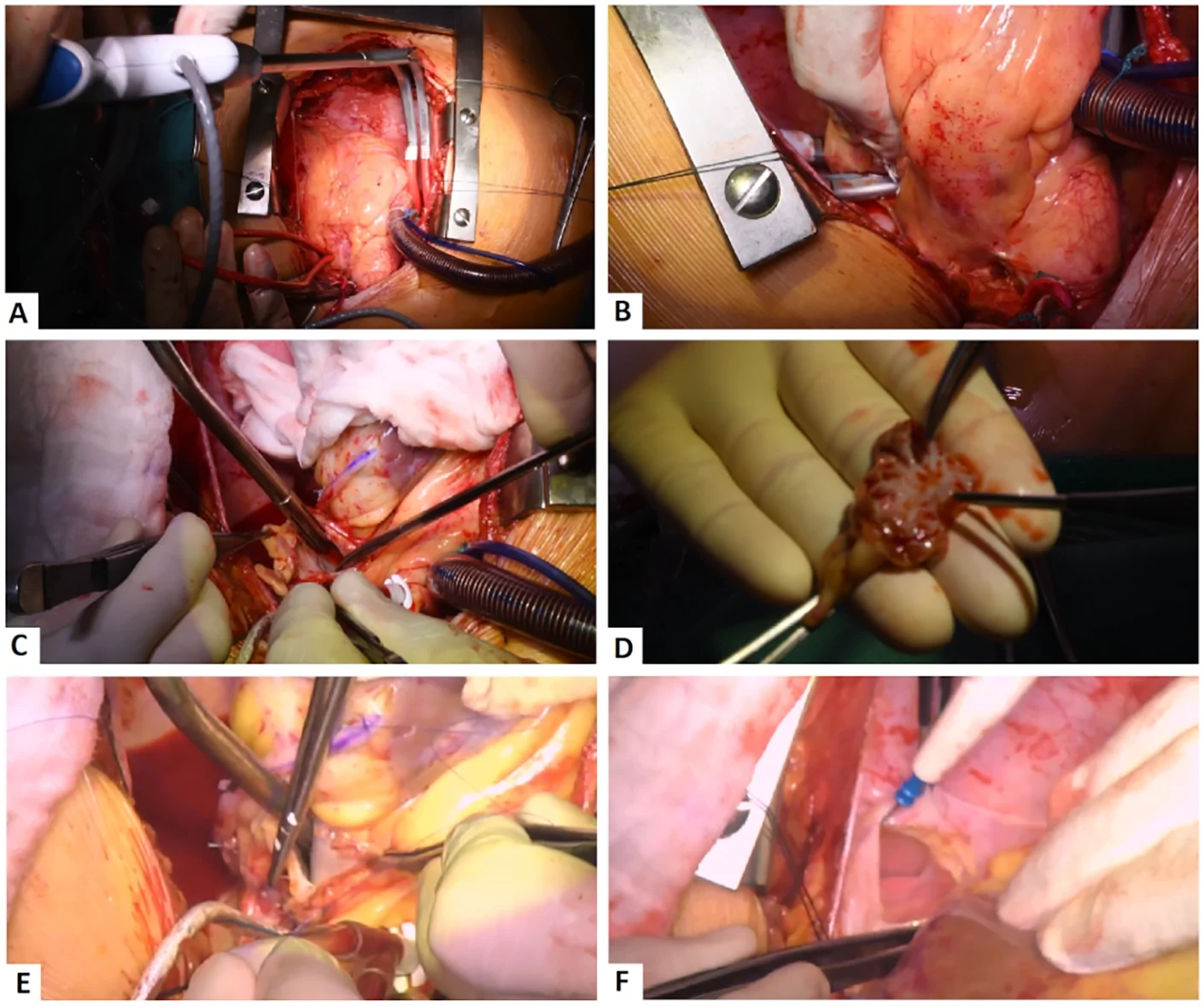
Ablation procedure performed during the initial cardiac surgery. **(A)** Pulmonary vein isolation and creation of a left atrial box lesion, facilitated by safe positioning of the magnetically attached clamp jaws along the red rubber guiding system. **(B)** The clamp jaws encompass the pulmonary veins together with the posterior wall of the left atrium. **(C,D)** Surgical amputation of the left atrial appendage. **(E)** Closure of the remaining left atrial opening. **(F)** Left-sided posterior pericardiotomy.

The AF ablation strategy was performed concomitantly with complete surgical coronary revascularization. The patient underwent four-vessel coronary artery bypass grafting, consisting of a left internal mammary artery to the left anterior descending artery and saphenous vein grafts to the posterior descending, obtuse marginal, and first diagonal branches. Cardiopulmonary bypass time was 175 min and aortic cross-clamp time 84 min. Moderate vasoactive support was required during weaning from cardiopulmonary bypass, consisting of dobutamine, norepinephrine, and vasopressin. No temporary mechanical circulatory support was ultimately required.

The postoperative course was uneventful, with extubation on postoperative day 1 and transfer to the ward on day 3. Echocardiography on postoperative day 7 demonstrated improved left ventricular function, with a left ventricular ejection fraction of 40%. A secondary prophylactic implantable cardioverter-defibrillator (ICD) was implanted uneventfully with borderline indication.

At discharge, the patient received a NOAC and guideline-directed heart failure therapy including beta-blockers. These therapies were continued during the 8-month follow-up.

ICD interrogation over the 8-month follow-up revealed sinus rhythm without atrial or ventricular arrhythmias (Figure 2).

**Figure 2:**
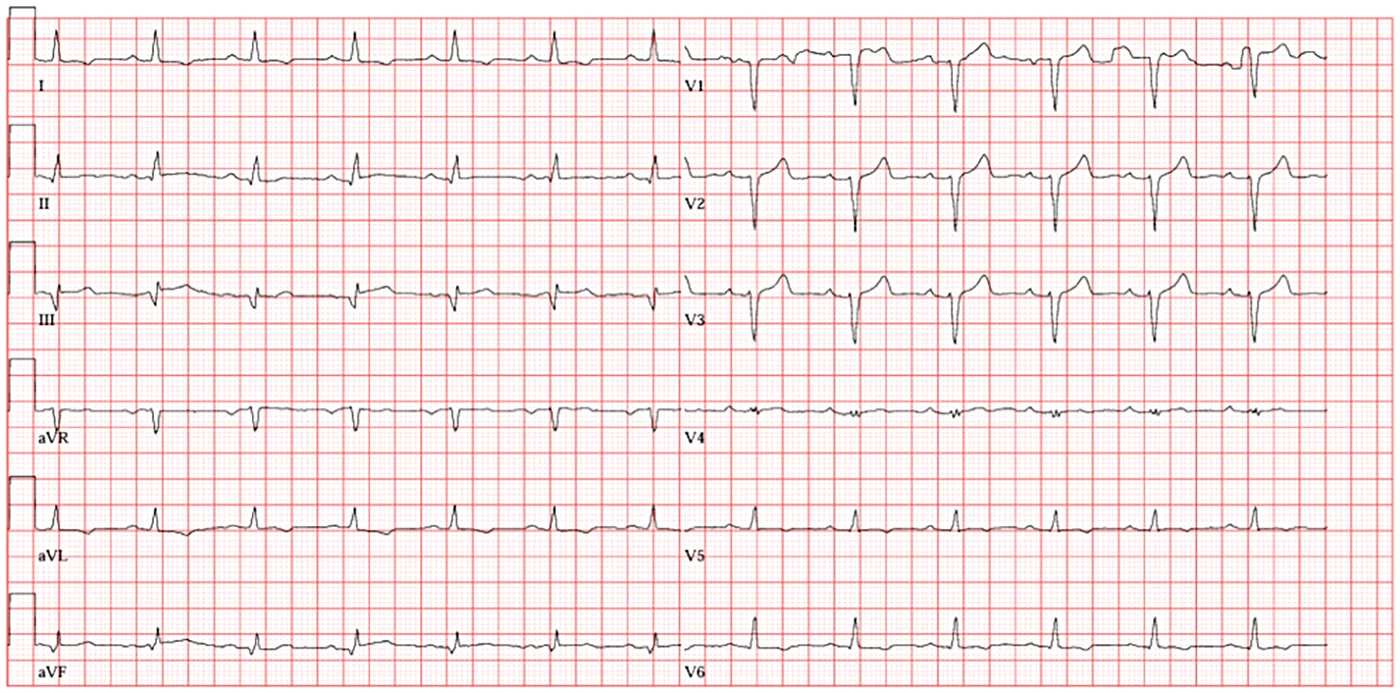
Electrocardiogram at eight-month follow-up after the ablation procedure, demonstrating sinus rhythm without conduction abnormalities.

The patient was satisfied with the combined surgical treatment of his coronary artery disease and atrial fibrillation. At follow-up, he reported no palpitations or recurrent symptoms, improved exercise tolerance, and a satisfactory postoperative recovery.

## Discussion

The present case describes our institutional standardized four-step approach to concomitant treatment of AF in patients undergoing non-atrioventricular valve cardiac surgery through a full sternotomy. Rather than representing four independent interventions, the protocol integrates complementary surgical strategies addressing different components of AF pathophysiology: pulmonary vein isolation and posterior left atrial isolation target the principal electrophysiological substrate, LAA exclusion addresses thromboembolic risk and potentially contributes to rhythm control, while LOM transection and posterior pericardiotomy may provide additional electrophysiological and postoperative benefits. The chronological sequence presented in the accompanying surgical video reflects the practical implementation of this integrated approach.

Pulmonary vein isolation remains the cornerstone of surgical treatment for AF, as the pulmonary veins represent the predominant source of ectopic triggers initiating paroxysmal AF. Bipolar radiofrequency technology enables the creation of reliable transmural lesions with real-time feedback on tissue conductance. In our protocol, circumferential isolation is extended to the posterior left atrium to create a box lesion, thereby addressing additional arrhythmogenic substrate beyond the pulmonary veins. This strategy appears particularly relevant in paroxysmal AF, in which pulmonary vein isolation with a box lesion has been associated with freedom from AF rates exceeding 80% at follow-up (6).

The second component, LAA management, complements the rhythm-directed part of the procedure by addressing the major source of thrombus formation in patients with AF. Contemporary guidelines therefore recommend surgical LAA exclusion in patients with AF undergoing cardiac surgery (7). We generally favor epicardial clip occlusion because of its reproducibility and high rates of complete exclusion (8). However, the present case illustrates that the method of LAA management may need to be individualized. Suspected thrombus formation and unfavorable anatomy caused by the adjacent coronary bypass graft to the proximal circumflex artery precluded safe clip placement. Surgical amputation with direct closure therefore provided an alternative, although bleeding from the LAA stump can represent a relevant limitation.

LOM transection represents an additional, less commonly addressed component of surgical AF treatment. The LOM contains myocardial sleeves, autonomic nerve fibers, and ganglionated plexi and has been implicated in both AF initiation and maintenance (9). Although division of the LOM is unlikely to provide sufficient substrate modification as an isolated intervention, its integration into a broader lesion set may interrupt potentially relevant autonomic and electrophysiological connections. Given its simplicity and limited additional operative burden, we consider LOM transection a pragmatic adjunct to pulmonary vein isolation rather than a stand-alone treatment.

Finally, posterior pericardiotomy extends the concept of AF prevention beyond direct atrial substrate modification. Originally introduced to reduce postoperative pericardial effusions and tamponade, randomized studies have subsequently demonstrated a reduction in postoperative AF following this procedure (10). Its incorporation into the protocol therefore provides a complementary postoperative preventive strategy without requiring additional atrial ablation.

Taken together, the four components address different and potentially complementary mechanisms involved in AF and its postoperative consequences. The standardized sequence provides a reproducible framework for concomitant AF treatment while preserving the flexibility to adapt individual components—particularly the technique of LAA exclusion—to patient-specific anatomy and intraoperative findings. The present case illustrates this principle: although the overall strategy followed a predefined sequence, the method of LAA management was modified according to the individual anatomical and clinical circumstances. Standardization, therefore, should not be understood as a rigid procedure, but as a structured framework supporting individualized surgical decision-making.

Surgical approaches to concomitant AF treatment vary considerably among centres and are influenced by AF phenotype, the concomitant procedure, surgical access, institutional expertise, and available ablation technology. The spectrum ranges from pulmonary vein isolation alone, with or without additional left atrial lesion sets and LAA exclusion, to the more extensive biatrial Cox-Maze procedure, which remains the most comprehensive surgical lesion set, particularly for persistent and long-standing persistent AF with advanced atrial substrate. However, the Cox-Maze procedure requires atriotomy and a more extensive lesion set (11, 12). Our approach occupies an intermediate position by combining epicardial pulmonary vein isolation with a posterior left atrial box lesion, LAA management, LOM transection, and left-sided posterior pericardiotomy. Importantly, the ablation component can be performed without left atriotomy or additional cross-clamp time, facilitating its integration into procedures in which left atrial access is otherwise not required. This may be particularly relevant for patients with paroxysmal or selected persistent AF in whom a less extensive lesion set is considered appropriate (6, 13). Conversely, the protocol should not be regarded as equivalent to a complete Cox-Maze procedure and may be less suitable for patients with persistent or long-standing persistent AF and extensive atrial substrate (1, 12, 14).

Another potential advantage of the protocol is its integration into a wide range of cardiac procedures without substantially altering the established surgical workflow. A predefined sequence of relatively straightforward steps may reduce variability in surgical practice and facilitate consistent consideration of concomitant AF treatment, particularly during coronary artery bypass grafting, aortic valve surgery, and ascending aortic procedures. Nevertheless, standardization should facilitate rather than replace clinical judgment, and the decision to apply the complete protocol should remain individualized according to AF phenotype, atrial substrate, anatomy, and the planned concomitant operation.

In conclusion, our institutional four-step AF protocol integrates complementary surgical strategies into a standardized and reproducible approach for patients undergoing non-atrioventricular valve cardiac surgery. By combining epicardial pulmonary vein and posterior left atrial isolation with LAA management, LOM transection, and left-sided posterior pericardiotomy, the protocol addresses different components of AF pathophysiology and its postoperative consequences without requiring left atriotomy or additional cross-clamp time. Its principal value lies in providing a structured framework that can be incorporated into established surgical workflows while allowing individual components to be adapted to patient-specific anatomy and AF characteristics. Further evaluation in larger patient cohorts is required to determine the rhythm and clinical outcomes associated with this approach and to define the patient populations in whom it may be most appropriate.

## Limitations

As a single-case observation, no conclusions can be drawn regarding comparative efficacy or impact on survival. The 8-month follow-up limits assessment of the long-term durability of the ablation. Further prospective studies and larger cohort analyses are required to validate the reproducibility, safety, and clinical benefit of this combined approach.

## Conclusion

Although concomitant surgical atrial fibrillation ablation carries a Class I recommendation for mitral valve surgery and a Class II recommendation for non-mitral valve surgery in current guidelines, its implementation in routine clinical practice remains variable. This case demonstrates that a standardized four-step epicardial ablation strategy can be feasibly integrated into coronary artery bypass surgery in a high-risk patient with severely reduced left ventricular function. By avoiding left atriotomy and minimizing procedural complexity, this approach may facilitate concomitant rhythm control without relevant impact on operative workflow. Further studies are required to evaluate its reproducibility and long-term clinical outcomes.

## Data Availability

The original contributions presented in the study are included in the article/Supplementary Material, further inquiries can be directed to the corresponding author.
